# Evidence-based indicators for the measurement of quality of primary care using health insurance claims data in Switzerland: results of a pragmatic consensus process

**DOI:** 10.1186/s12913-018-3477-z

**Published:** 2018-09-27

**Authors:** Eva Blozik, Oliver Reich, Roland Rapold, Martin Scherer, Swiss Quality Indicator for Primary Care (SQIPRICA) Working Group, Sima Djalali, Felix Huber, René Kühne, Jan von Overbeck, Thomas Rosemann, Felix Schneuwly, Martin Scherer, Oliver Senn, Daniel Tapernoux

**Affiliations:** 1 Department of Health Sciences, Helsana Group, P.O. Box, Zürich, Switzerland; 2 Department of General Practice/Primary Care, University Medical Center Hamburg-Eppendorf, Hamburg, Germany; 3 Division of General Practice, University Medical Centre Freiburg, Freiburg, Germany

**Keywords:** Quality indicator, Quality assessment, Quality measurement, Claims data, Health insurance, Evidence-based, Consensus process

## Abstract

**Background:**

The level of quality of care of ambulatory services in Switzerland is almost completely unknown. By adapting existing instruments to the Swiss national context, the present project aimed to define quality indicators (QI) for the measurement of quality of primary care for use on health insurance claims data. These data are pre-existing and available nationwide which provides an excellent opportunity for their use in the context of health care quality assurance.

**Methods:**

Pragmatic 6-step process based on informal consensus. Potential QI consisted of recommendations extracted from internationally accepted medical practice guidelines and pre-existing QI for primary care. An independent interdisciplinary group of experts rated potential QI based on explicit criteria related to evidence, relevance for Swiss public health, and controllability in the Swiss primary care context. Feasibility of a preliminary set of QI was tested using claims data of persons with basic mandatory health insurance with insurance at one of the largest Swiss health insurers. This test built the basis for expert consensus on the final set of QI.

**Results:**

Of 49 potential indicators, 23 were selected for feasibility testing based on claims data. The expert group consented a final set of 24 QI covering the domains general aspects/ efficiency (7 QI), drug safety (2), geriatric care (4), respiratory disease (2), diabetes (5) and cardiovascular disease (4).

**Conclusions:**

The present project provides the first nationwide applicable explicit evidence-based criteria to measure quality of care of ambulatory primary care in Switzerland. The set intends to increase transparency related to quality and variance of care in Switzerland.

## Background

A broad spectrum of initiatives aim to increase the quality of primary care in Switzerland [1–3]. These initiatives include a variety of different approaches such as certification measures [4–6], in-house medical guideline development or quality circles [7] that vary in terms of regional spread, objectives, target population, and evidence basis. Despite this wide range of projects, the level of quality of care of ambulatory services in Switzerland is almost completely unknown [8]. This is especially paradoxical, as the Swiss health insurance act (Art. 22a Krankenversicherungsgesetz) requires the collection of quality information in the context of Swiss basic mandatory health insurance. Since several years, the Swiss Federal Office of Public Health is working on strategies to realise these requirements, which are practically disregarded within the current collective tariff agreements such as TARMED, the tariff system of ambulatory medical procedures in Switzerland [9]. However, results or at least initiatives addressing this issue are still lacking. Therefore, the topic of developing defined and feasible approaches for national quality assurance in ambulatory care are of great political and practical relevance.

According to the Institute of Medicine (IOM) report, *To Err Is Human*, most errors in health care result from inefficient and variable processes, changing case mix of patients, inconsistencies in health service reimbursement systems, differences in provider education and experience, and numerous other factors [10]. However, quality improvement is not possible without quality measurement [11].

Quality indicators (QI) are measurable items designed to assess, compare, and improve quality of health services [12]. So far, there are no QI for measurement of quality of care of primary care outpatient services established for application within the Swiss healthcare setting, despite some small certification programs, on a voluntary basis.

Although there are well-known limitations, health insurance claims data of mandatory basic health insurance provide a valuable opportunity for their use in the context of health care quality assurance. These real world data are pre-existing, nationwide available, and they link information on individual patients with information on healthcare providers, settings and health plans. Therefore, they allow for both cross-sectional and longitudinal evaluations on different levels from the individual patient to the system level.

As Switzerland is lagging behind other European countries with respect to quality measurement in the ambulatory sector [13–16], the present project aims to define a set of evidence-based QI for the measurement of quality of primary care [17, 18]. To increase applicability without the barrier of huge preceding investments the QI are intended to be used on Swiss health insurance claims data. The present study responds to political discussions about how to increase transparency related to quality of ambulatory care in Switzerland by pragmatically combining pre-existing evidence-based methods with local expertise.

## Methods

### Context of the study

Health insurance is mandatory for all persons residing in Switzerland. The basic health insurance package is the same in the entire country and includes all outpatient or hospital medical treatments deemed appropriate, medically effective, and cost-effective. Supplementary hospital insurance in Switzerland can be purchased, if individuals wish further comfort of a semiprivate or private ward or treatment in another canton for personal reasons. There are about 60 insurance companies providing basic health coverage in Switzerland, and they offer a range of different premiums and health plans from which Swiss residents are free to choose [19]. Registering with a GP is generally not required, and residents insured in the standard insurance plan have free choice among mostly self-employed GPs. However, persons are free to enrol in managed care plans (e.g. integrated care plans, telephone triage plans, capitated and non-capitated plans) in which they need to contact a specific primary care provider before seeking care with other healthcare providers. In 2016, there were 0,95 generalist physicians per 1000 inhabitants in the ambulatory sector [20]. Primary (and specialist) care tends to be physician-centered, with nurses and other health professionals playing a relatively small role [21]. The present project is an initiative of the health services research department of Helsana Group. Helsana is one of the largest Swiss health insurances covering about 15% of the Swiss population from all parts of the country.

### Study protocol

**Fig. 1:**
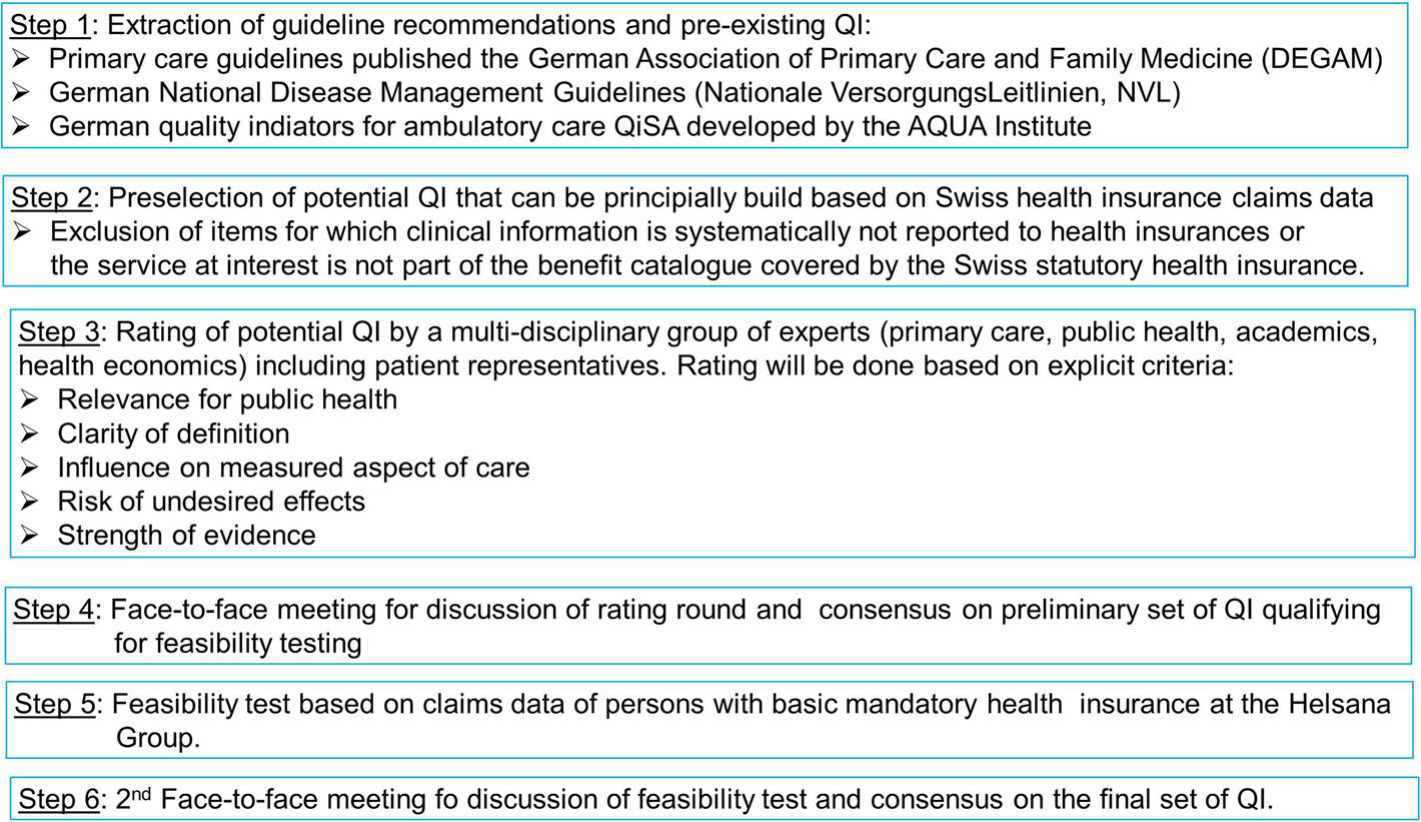
Study protocol

The process of defining suitable QI consisted of several steps (Fig. 1). In Switzerland, there is no formal or national process or institution responsible for summarizing and operationalization of evidence for quality improvement purposes. National guidelines for primary care in Switzerland are lacking. However, the Swiss and the German health system have many structural similarities such as the existence of a social health insurance system, the public/ private mix of health service providers, the choice between various competing health insurances, and financing of health care costs by premiums, public funds, and co-payments. Moreover, there are no linguistic barriers as both countries are part of the German-speaking area. Therefore, the present project based on recommendations from guidelines of the German association of primary care and family medicine (Deutsche Gesellschaft für Allgemeinmedizin und Familienmedizin, DEGAM) and the German National Disease Management Guidelines (Nationale VersorgungsLeitlinien, NVL) and on QISA (QI for primary care, developed by the AQUA Institute) indicators. Development of guidelines or QI followed an established clearly defined and internationally acknowledged methodology [22–24].

In a first step, we extracted all recommendations for or against specific medical interventions of all currently existing DEGAM and NVL guidelines and all QISA. Not all recommendations and QI extracted can be constructed using the information that is available in health insurance claims. Therefore, in a second step, this list of potentially eligible items for QI was checked for operationalisability on Swiss health insurance claims data. In a third step, a multidisciplinary group of 9 independent experts (Swiss Quality Indicator for Primary Care (SQIPRICA) Working Group) from primary care, public health, and health economics including patient and consumer representatives rated the list of potential QI. Criteria for rating were relevance for public health, clarity of definition, influence on measured aspect of care, risk of undesired effects, and strength of evidence. The rating process was derived from the methodology used for development of QI in the context of national disease management guidelines in Germany [22]. Influenceability was defined as the potential of a GP to modify care. For example, interventions generally done in the hospital setting were considered to be not influenceable. Experts were asked to rate the potential indicators according to a 4 point Likert scale (1 = incorrect; 2 = rather incorrect; 3 = rather correct; 4 = fully correct). For the aspect risk of undesired effects, they were asked to answer yes or no. The aim of including the expert group was to ensure that the resulting QI were calculable, relevant, and influenceable by primary care within the Swiss healthcare system.

As a fourth step, there was a face-to face-meeting of the project team and the expert group. The objective of that workshop was to discuss discrepant rating results and principal difficulties, to converse the strengths and limitations of claims data in this context, and to reach consensus on a preliminary set of QI qualifying for a first practical test. For preparation of the workshop, the experts received a descriptive analysis of the rating results (mean, median, range of Likert scale ratings) for each candidate QI by criterion (i.e. relevance for public health, clarity of definition, influence on measured aspect of care, risk of undesired effects, and strength of evidence). We did not apply any cut-points for exclusion based on Likert score rating results. Therefore, the rating results of all potential indicators included in step 3 were made visible to the expert group members and were used as a basis for discussion in the group.

The fifth step was a feasibility test. This was done using claims data of 950′000 adult persons with basic mandatory health insurance in the year 2013. The patient-level database included information on socio-demographics, health insurance status, prescribed drugs, health care utilization and its associated costs as well as hospital discharge information and the date of death. In this sample, the proportion of persons presenting with the QI at interest was calculated, stratified by socio-demographic (age class, sex, resident in French or Italian speaking canton, amount of individually eligible annual deductible, voluntary choice of a managed care health plan) and clinical (cancer, cardiovascular disease, psychological disorders as measured using pharmaceutical cost groups (PCG)) characteristics [25]. Inefficient me-too medications were operationalized based on the list published by Fricke & Klaus [26]. Potentially Inappropriate Medication (PIM) are pharmaceuticals associated with an increased risk for adverse drug reactions in older persons that should be avoided whenever possible, according to expert consensus. PIM were defined based on the Beers criteria and the PRISCUS list [27, 28]. Each active agent and combinations from these lists were attributed to one or more ATC Anatomical Therapeutic Chemical Classification System (ATC) codes [29]. The definition of PIM variables accounted for the fact that certain medications were considered inappropriate only above a certain dose or for long-term use. Analyses were performed using the statistical package R, version 3.2.0 (R Foundation for Statistical Computing, Vienna, Austria).

As a final (sixth) step a second workshop with the expert group was performed to discuss the results of the feasibility test, to receive recommendations for adaptation and to reach consensus about the final set of QI.

### Ethical approval

The analysis complied with the Swiss Federal Law on data protection. All data were anonymized and de-identified prior to the performed analysis to protect the privacy of patients, physicians, and hospitals. According to the national ethical and legal regulation, an ethical approval was not needed because the data were retrospective, pre-existing, and de-identified. Since data was anonymized, no consent of patients was required.

## Results

We extracted guideline recommendations and QI from 12 National Disease Management Guidelines, 12 QiSA indicator sets and 8 DEGAM primary care S3 guidelines.

We excluded duplicates, services that are not part of the basic mandatory health insurance package in Switzerland and measures that cannot be mapped using claims data such as details of clinical processes, decision making, or communication that are not relevant for reimbursement. A list of 49 potential QI was sent to the expert group for rating of relevance for public health, clarity of definition, influence on measured aspect of care, risk of undesired effects, and strength of evidence. Overall, there were few discrepancies related to the rating across the group. All potential QI were assigned high values for the aspect “relevance for public health” (mean and median 3 = “rather correct”).

Table 1 lists those indicators that were – according to expert consensus - rated inadequate for use as a QI in the Swiss healthcare setting and were therefore excluded. Reasons for exclusion were the indicator not being influenceable by primary health care providers, being irrelevant for Swiss primary healthcare due to medical practice, medication market or patient population, or the indicator not being calculable due to lack of clinical information in health insurance claims data. These concerns were reflected in consistently low Likert scores for “influence on measured aspect of care” and “risk of undesired effects” for all those potential QI listed in Table 1. The workshop resulted in a set of 23 preliminary indicators qualifying for the feasibility test covering the domains general aspects/ efficiency (7 QI), drug safety (2), geriatric care (4), respiratory disease (3), diabetes (4) and cardiovascular disease (3).

**Table 1:** Results of the first workshop: rated inappropriate for use as a QI in the Swiss healthcare setting

Category	Potential indicator	Reason
General aspects, efficiency	Proportion of patients enrolled in health plans per region	Not related to quality of primary care
General aspects, efficiency	Proportion of hospitalisations for interventions that can be adequately done in the ambulatory setting	Measures quality of hospital care/ hospital processes
General aspects, efficiency	Number of hospitalisations per 1000 persons	Rather a measure of hospital processes, and density parameters than of primary care
General aspects, efficiency	Proportion of patients receiving medication therapy	No clear indicator of quality. Not specific to primary care.
General aspects, efficiency	Share of prescriptions of new me-too medications of the total market	A current classification of me-too medications is not available for Switzerland
Respiratory disease	Proportion of patients with asthma or COPD receiving combinations of reproterol & cromoglicinic acid	reproterol and cromoglicinic acid are not on the Swiss market/ use is very unusual
Respiratory disease	Proportion of patients with asthma or COPD receiving, die N-acetylcystein, ambroxol or myrtol for elimination of secret	Medications are not on the Swiss market/ use is very unusual
Respiratory disease	Proportion of patients with COPD receiving pneumococcal vaccination	Asthma and COPD cannot be differentiated in the claims dataset because ambulatory diagnoses are lacking
Respiratory disease	Proportion of pregnant women with incident therapy with leukotriene receptor antagonists	Relatively small number of cases, therefore not suitable for large scale measurement
Respiratory disease	Proportion of pregnant women with incident specific immunotherapy	Relatively small number of cases, therefore not suitable for large scale measurement
Respiratory disease	Proportion of children/ teenagers with asthma receiving oral beta-2-sympathomimetics in acute situations	Clinial information is missing
Respiratory disease	Proportion of patients with asthma receiving inhalative medication	Identification of patients is based on medication. Therefore, no meaningful interpretation of results possible.
Respiratory disease	Proportion of patients with asthma with long term inhalative corticosteroids	Asthma and COPD cannot be distinguished using claims data. Identification of patients is based on medication. Therefore, no meaningful interpretation of results possible.
Cardiovascular disease	Proportion of patients with heart failure receiving laboratory control of electrolytes and renal function semi-yearly	Population cannot be determined because ambulatory diagnoses are lacking in Swiss health insurance claims
Cardiovascular disease	Proportion of patients after coronary stent implant receiving triple therapy (ASS + Clopidogrel + Anticoagulation)	Measures quality of care of cardiologists/ interventional cardiologists (as opposed to primary care)
Cardiovascular disease	Proportion of patients after aortocoronary Bypass / acute coronary syndrome and Anticoagulation only	Measures quality of care of cardiologists/ interventional cardiologists (as opposed to primary care) Measures quality of hospital care
Cardiovascular disease	Proportion of patients after coronary bypass receiving multidisciplinary rehabilitation	No coherent way of accounting of rehabilitation services in Switzerland, no information about multidisciplinarity
Cardiovascular disease	Proportion of ambulatory patients with laboratory test for BNP und NT-proBNP	Recommendation is not clear enough.
Depression	Proportion of patients resistant to depression treatment receiving augmentation of antidepressants with carbamazepine, lamotrigine, pindolol, valproate, dopamine agonists, psychostimulants, thyroid hormone or other hormones	Clinical information is missing
Diabetes mellitus	Proportion of patients with pain in diabetic neuropathy treated with traditional nonsteroidal antiphlogistics	Clinical information is missing
Diabetes mellitus	Proportion of patients with pain in diabetic neuropathy treated with selective Cox-2 inhibitors	Clinical information is missing

The results of the feasibility test were discussed in a second face-to face meeting of the project team and the expert group. According to the experts assessment, the feasibility test revealed that it was possible to operationalize all preliminary indicators, and all indicators were sensitive to age, gender, PCG, and to characteristics of health insurance. However, based on discussion related to actual public health needs, applicability and influenceability, the expert committee decided to modify the preliminary set as follows: 1 asthma indicator was dropped because of limited control of primary care physician. The definition and number of indicators related to diabetes mellitus were made more consistent with recent Swiss real-life evidence [30–32]. In addition, the indicators relating to care for patients with cardiovascular disease were made more specific by investigating therapy with statins and ASS separately in two different patient subgroups (i.e. patients after myocardial infarction and patients after stroke). Based on informal consensus, the experts passed a final set of 24 QI including 7 QI measuring general aspects/ efficiency, 2 QI assessing drug safety, 4 QI related to geriatric care, and 11 QI measuring the management of highly prevalent chronic diseases (2 respiratory disease, 5 diabetes mellitus, 4 cardiovascular disease) (Table 2).

**Table 2:** Final set of quality indicators

Number	Category	Subject	Nominator	Denominator	Comments
1	General aspects, efficiency	Number of emergency hospital admissions per 1000 insured persons	Number of emergency hospital admissions	Number of insured persons	Measure is sensitive to density of health care providers, culture, socioeconomics and other factors not influenceable by primary care
2	General aspects, efficiency	Medication costs per insured person	Sum of gross medication costs per insured person irrespective of the prescriber	Number of insured persons	Measure is sensitive to case mix
3	General aspects, efficiency	Costs per daily dose in specific ATC groups relevant in primary care	Sum of gross medication costs	Sum of daily doses	Practical implementation depends on quality of medication master data of claims insurance database. Might be more easily implementable for few most important ATC groups^a^
4	General aspects, efficiency	Proportion of prescriptions of generics	Sum of prescriptions of generics	Sum of prescriptions of generics eligible medication	The original QISA indicator measures the share of generics in the overall market. As this depends on the market of generics and on approval policy and is not
					influenceable by primary care, the group decided to specify the indicator.
5	General aspects, efficiency	Proportion of prescriptions of inefficient me-too medications	Sum of prescriptions of medications listed on corresponding lists^b^	Sum of all medication prescriptions	Lists need adaptation to Swiss medication market
6	General aspects, efficiency	Number of different primary care physicians consulted by an individual insured person	Number of different primary care physicians consulted per insured person	Number of insured persons with at least 1 primary care physician consultation	Interval of interest needs to be determined. Persons enrolled in managed care health plans are presumed to have a value of 1 or marginally higher than 1.
7	General aspects, efficiency	Number of different specialist physicians consulted by an individual insured person	Number of different specialist physicians consulted per insured person	Number of insured persons with at least 1 physician consultation	Interval of interest needs to be determined. Number of persons enrolled in managed care plans is presumed to be lower as compared to persons enrolled in plans without collaboration/ coordination of care.
8	Drug safety	Number of prescriptions of anxiolytics, sedatives or hypnotics	Number of prescriptions of anxiolytics, sedatives or hypnotics per quarter year	Number of insured persons with at least 1 medication prescription per quarter year	The original QISA indicator measures the proportion of persons receiving more than 30 DDD of persons receiving anxiolytics, sedatives, or hypnotics. Swiss health insurance claims provide currently insufficient detail/data quality to measure DDD of anxiolytics, sedatives, and hypnotics on a routine basis. The expert group recommended therefore to use number or prescriptions as crude but still informative measure.
9	Drug safety	Number of prescriptions of non-steroidal anti-inflammatory drugs (NSAIDs)	Number of NSAIDs prescriptions per quarter year	Number of insured persons with at least 1 NSAIDs prescription per quarter year	The original QISA indicator measures the proportion of persons receiving more than 75 DDD of persons receiving NSAIDs. Swiss health insurance claims provide currently insufficient detail/data quality to measure DDD of NSAIDs on a routine basis. The expert group recommended therefore to use number or prescriptions as crude but still informative measure.
10	Geriatric care	Proportion of insured persons aged 65 years or older with polymedication	Sum of insured persons aged 65 years or older with 5 or more different medication prescriptions per quarter year	Sum of insured persons aged 65 year or older with at least 1 medication prescription (related to quarter year)	Based on difference of ATC codes
11	Geriatric care	Proportion of insured persons aged 65 years or older with prescription of potential inappropriate medications (PIM)	Sum of insured persons aged 65 years or older with PIM prescriptions per quarter year	Sum of insured persons ages 65 years or older with at least 1 medication prescription (related to quarter year)	Based on ATC codes and PRISCUS list and Beers criteria
12	Geriatric care	Proportion of insured persons aged 65 year or older with reimbursed influenza vaccination	Sum of insured persons aged 65 year or older with reimbursed influenza vaccination per year	Sum of insured persons aged 65 year or older per year	Evidence is unclear, currently controverse discussions. Subject to patient preferences and shared decision making.
13	Geriatric care	Proportion of insured persons aged 65 year or older with at least one chronic condition who were hospitalised for fracture near the pelvic joint	Sum of insured persons aged 65 year or older with at least one chronic condition (presence of at least 1 PCG) who were hospitalised for fracture near the pelvic joint	Sum of insured persons aged 65 year or older per year	The original QISA indicator refers to persons older than 70 years. The expert committee preferred to specify a potentially frail older population based on comorbidity. For identification of hospitalization due to for fracture near the pelvic joint, SwissDRG codes or ICD codes can be used. DRG codes are less precise.
14	Respiratory disease	Proportion of insured persons receiving long term therapy of systemic corticosteroids	Sum of insured persons with the Pharmacy Cost Group “respiratory disease” receiving systemic corticosteroids in two sequential quarter years	Sum of insured persons with the Pharmacy Cost Group “respiratory disease”	ATC H02. When interpreting the data it should be considered that currently, asthma and COPD cannot clearly be distinguished based on Swiss health insurance data.
15	Respiratory disease	Disease-specific hospitalisation rate of insured persons with the Pharmacy Cost Group “respiratory disease”^c^	Sum of insured persons with the Pharmacy Cost Group “respiratory disease” hospitalised because of complications of respiratory disease	Sum of insured persons with the Pharmacy Cost Group “respiratory disease”	Low controllability by primary care physician. Might be influenceable by efficient therapy. For identification of hospitalization due to asthma or COPD, SwissDRG codes or ICD codes can be used. DRG codes are less precise.
16	Diabetes mellitus	Proportion of insured persons with antidiabetic medication receiving which HbA1c controls (number of controls per year)	Sum of insured persons with the Pharmacy Cost Group “diabetes mellitus” for which HbA1c controls were reimbursed per year	Sum of insured persons with the Pharmacy Cost Group “diabetes mellitus”	Current guidelines recommend HbA1c controls at least each or every second quarter year. The expert group recommends stratified measurement of 1, 2, 3 and 4 HbA1c controls per year. Diabetic patients without antidiabetic medication will be missed.
17	Diabetes mellitus	Proportion of insured persons with antidiabetic medication receiving which an ophthalmologic control within 15 months	Sum of insured persons with the Pharmacy Cost Group “diabetes mellitus” receiving which an ophthalmologic control within 15 months	Sum of insured persons with the Pharmacy Cost Group “diabetes mellitus”	Current guidelines slightly vary in recommended intervals (between 1 and 2 years). The expert group recommends 15 months. An interval of 2 years would be clinically reasonable and calculation would be easier.
18	Diabetes mellitus	Hospitalisation rate of insured persons with antidiabetic medication	Sum of insured persons with the Pharmacy Cost Group “diabetes mellitus” with at least 1 hospitalisation per year	Sum of insured persons with the Pharmacy Cost Group “diabetes mellitus” per year	Good quality primary care (information, patient self-management skills, medication and non-medical therapies, coordinated care etc.) prevents hospitalisation of diabetic patients. Therefore, focusing non-disease-specific hospitalisations is reasonable.
19	Diabetes mellitus	Proportion of insured persons with antidiabetic medication receiving control of lipid values per year	Sum of insured persons with the Pharmacy Cost Group “diabetes mellitus” for which control of lipid values was reimbursed per year	Sum of insured persons with the Pharmacy Cost Group “diabetes mellitus” per year	
20	Diabetes mellitus	Proportion of insured persons with antidiabetic medication receiving control of kidney values per year	Sum of insured persons with the Pharmacy Cost Group “diabetes mellitus” for which control of kidney values was reimbursed per year	Sum of insured persons with the Pharmacy Cost Group “diabetes mellitus” per year	
21	Cardiovascular disease	Proportion of insured persons with hospitalization for myocardial infarction receiving acetylsalicylic acid (ASS)	Sum of insured persons with hospitalization for myocardial infarction receiving ASS per year	Sum of insured persons with hospitalization for myocardial infarction per year	Extrapolating the patient’s medical long term history based on Swiss health insurance data is limited for technical reasons, changes in legislation (no diagnostic information from hospitals before 2012) and the right to change the health insurance every year. Should be operationalized pragmatically, e.g. proportion of insured persons with hospitalization for myocardial infarction in preceding year receiving acetylsalicylic acid in the year following the event.
22	Cardiovascular disease	Proportion of insured persons with hospitalization for myocardial infarction receiving statins	Sum of insured persons with hospitalization for myocardial infarction receiving statins per year	Sum of insured persons with hospitalization for myocardial infarction per year	As mentioned above.
23	Cardiovascular disease	Proportion of insured persons with hospitalization for stroke receiving ASS	Sum of insured persons with hospitalization for stroke receiving ASS per year	Sum of insured persons with hospitalization for stroke per year	As mentioned above.
24	Cardiovascular disease	Proportion of insured persons with hospitalization for stroke receiving statins	Sum of insured persons with hospitalization for stroke receiving statins per year	Sum of insured persons with hospitalization for stroke per year	As mentioned above.

All indicators should be stratified by age, gender, and - if feasible - by comorbidity

^a^ATC groups of medications both relevant in primary care and with sufficient data quality were: proton pump inhibitors (A02BC), selective betablockers (C07AB), selective serotonin reuptake inhbitors (SSRI) (N06AB), bisphosphonates (M05BA), triptanes (N02CC), dihydropyridine type calcium channel blockers (C08CA), oral antidiabetics (A10B), antidepressants (N06A), and systemic corticoids (H02A)

^b^lists such as the list published by Fricke & Klaus [26]

^c^includes patients with asthma or COPD

## Discussion

The present project provides the first evidence-based nationwide measures for quality of primary ambulatory care in Switzerland applicable on pre-existing data. The consensus process resulted in 24 indicators that are - in principle - ready for use in a broad variety of contexts. For example, 4 indicators for quality care of diabetes patients have been recently included in pay-for-performance (P4P) contracts between networks of primary care physicians and a Swiss health insurance [33]. On a higher level, the proposed QI help to increase transparency related to the level of and awareness for variance of quality of primary care in Switzerland. In addition, they may build the basis for quality assurance projects of health service providers. Therefore, such indicators may also be helpful for decision making of all stakeholders in the Swiss health system.

Recently, Ebert et al. published the Swiss Primary Care Active Monitoring (SPAM) instrument consisting of 56 indicators related to the organization of primary care in Switzerland. SPAM tool aims to support a better understanding of the Swiss PC system’s performance and effectiveness on a meta level [34]. It addresses collaboration and coordination of stakeholders, access, and supply of services and care providers. But does not focus on quality in various clinical situations as our indicators do. Thus, most SPAM aspects are not included in reimbursement information and can thus not be measured based on health insurance claims data. Therefore, SPAM complements the QI proposed by our group. Measuring quality both on the individual patient level and on a health system level might multiply insight in potential ways to improve quality of ambulatory primary care in Switzerland. The QI identified in the present study provide the opportunity to compare physician networks or even individual health service providers and therefore expand the operational conclusions that may be drawn from quality measurement.

Several limitations need to be considered. Firstly, we recruited a quasi-representative sample of experts for adapting international evidence to the Swiss healthcare setting. Therefore, the participants were not official representatives of stakeholder institutions but were selected based on their expertise related to primary care and/ or the Swiss health system. Secondly, the project was done from the perspective of Swiss mandatory basic health insurance. Therefore, services usually performed outside of the basic health insurance package were systematically not addressed in this project (e.g. services of supplementary insurance, over the counter medication, health-related life style, health promotion and prevention). However, the Swiss Swiss mandatory basic health insurance covers a very broad spectrum of all services needed for management of illness, accidents, and motherhood deemed to be effective, appropriate. and cost-efficient [35]. Thirdly, as our aim was to define QI for application on health insurance claims, we had to systematically exclude all aspects of quality that were not relevant for billing in the system of basic health insurance in Switzerland. Therefore, quality as reflected in satisfaction, communication, information, decision-making, or clinical results that impact the provision of health services at interest need to be addressed elsewhere. Fourthly, evidence-based QI can only be as good as the underlying evidence. Therefore, several aspects might be systematically under-or overrepresented depending on the presence or absence of evidence in certain clinical areas. Finally, data for feasibility testing came from a single health insurance, and results might differ when including data from other health insurances. However, the Helsana Group covers about 15% of the Swiss population, and the representative nature of the data has repeatedly been shown before [36, 37].

The main strength of the project is the pragmatic methodology in response to current public debates on quality assurance in ambulatory care. The present study combined pre-existing evidence-appraisal from devoted institutions in Germany, internationally accepted methods for QI development, local expertise, and the pre-existing nature of health insurance claims data. Moreover, both the patient and the consumer perspective were represented in the expert group as considered the gold standard for the development of QI [38, 39].

The present study has implications for future research. First, use of the proposed indicators needs to be evaluated. Specifically, future studies should assess if and how behaviour of physicians, frequency of unwarranted events such as hospitalisations, or costs change after introduction of P4P contracts. This is especially relevant since the evidence for P4P is not yet fully clarified, and international experiences are discussed controversially [40, 41].

Secondly, variance of quality across regions, settings, health insurance plans and patient groups need to be explored. Thirdly, an evidence-based instrument needs continuous update, evaluation, and continual adaptation [42]. For example, a current research project aims to develop QI for multimorbidity [43], and such indicators might be suitable for adaptation to the Swiss context and for future integration in the present set of QI.

In practice, QI constructed upon health insurance claims may provide the impetus to increase efforts for more quality in Swiss primary healthcare, to differentiate incentives for health care providers, and to increase quality competition across health care providers, health plans, and health insurances. The present QI may build the basis for the implementation of models that fit the Swiss needs.

## Conclusions

Based on pre-existing foreign clinical practice guidelines and QI and on an informal expert consensus process we identified a broad set of QI for the measurement of quality of primary care in Switzerland that can be applied on nation-wide available health insurance claims data. Implementation of these indicators needs to be evaluated so that this set of QI can be continuously ameliorated and expanded. Local evidence related both to the level of quality of primary care and to the positive and negative effects of implementation of QI is urgently needed.

## Abbreviations

ASS: Acetylsalicylic acid
ATC: Anatomical therapeutic chemical classification system
COPD: Chronic obstructive pulmonary disease
DDD: Defined daily dose
NSAID: Nonsteroidal anti-inflammatory drug
PCG: Pharmacy cost group
PIM: Potentially inappropriate medication
QI: Quality indicator

## References

[1] Senn N, Ebert ST, Cohidon C (2016). Obsan Bulletin 11/2016: Die Hausarztmedizin in der Schweiz – Perspektiven. Analyse basierend auf den Indikatoren des Programms SPAM (Swiss Primary Care Active Monitoring).

[2] Schweizer Forum für Intergrierte Versorgung fmc: Neue Versorgungsmodelle für die medizinische Grundversorgung. Bericht der Arbeitsgruppe “Neue Versorgungsmodelle für die medizinische Grundversorgung” von GDK und BAG. 2012. https://www.fmh.ch/files/pdf13/versorgungsmodelle_d.pdf. Accessed 10 Feb 2018.

[3] Chmiel C, Bhend H, Senn O, Zoller M, Rosemann T (2011). The FIRE project: a milestone for research in primary care in Switzerland. Swiss Med Wkly.

[4] Stiftung EQUAM (2018). Unser Angebot.

[5] Mehrfacharzt: Die Zertifizierung als “MehrFachArzt” oder “MehrFachÄrztin”. 2018. http://www.mehrfacharzt.ch/fuer-hausaerzte/zertifizierung/. Accessed 10 Feb 2018.

[6] QBM-Stiftung: QBM – von Ärzten für Ärzte. 2018. https://www.qbm-stiftung.ch/qbm.html. Accessed 10 Feb 2018.

[7] Berchtold P, Schmitz C, Maier J (2012). Obsan Report 51: Guidelines in Schweizer Ärztenetzen. Entwicklung und Bedeutung.

[8] Meyer K (2010). Gesundheit in der Schweiz – Nationaler Gesundheitsbericht 2008.

[9] Swiss Federal Office of Public Health: Quality Assurance. 2018. https://www.bag.admin.ch/bag/en/home/themen/versicherungen/krankenversicherung/krankenversicherung-qualitaetssicherung.html. Accessed 10 Feb 2018.

[10] Institute of Medicine (1999). To err is human: building a safer health system.

[11] Lester H, Roland M, Smith P, Mossialos E, Papanicolas I, Leatherman S (2009). Performance measurement in primary care. Performance Measurement for Health System Improvement.

[12] Campbell SM, Kontopantelis E, Hannon K, Burke M, Barber A, Lester HE (2011). Framework and indicator testing protocol for developing and piloting quality indicators for the UK quality and outcomes framework. BMC Fam Pract.

[13] Swiss Federal Office of Public Health: Comparisons and Analyses of Health Systems. 2018. https://www.bag.admin.ch/bag/en/home/themen/internationale-beziehungen/internationalegesundheitsthemen/comparaisons-analyses-systemes-sante.html. Accessed 19 April 2018.

[14] Swiss Federal Office of Public Health: Ein Netzwerk für mehr Qualität in der Gesundheitsversorgung. 2018. https://www.bag.admin.ch/bag/de/home/themen/versicherungen/krankenversicherung/krankenversicherung-revisionsprojekte/netzwerk-qualitaet-gesundheitsversorgung.html. Accessed 19 April 2018.

[15] Limb M (2013). OECD finds some countries are too restrictive about sharing personal data. BMJ.

[16] Swiss Academy of Medical Sciences. Stärkung der Versorgungsforschung in der Schweiz. Swiss Academies Reports 2014; 9(1). https://www.samw.ch/de/Publikationen/Positionspapiere.html. Accessed 19 April 2018.

[17] Cohidon C, Cornuz J, Senn N (2015). Primary care in Switzerland: evolution of physicians’ profile and activities in twenty years (1993–2012). BMC Fam Pract.

[18] Tandjung R, Hanhart A, Bärtschi F, Keller R, Steinhauer A, Rosemann T, Senn O (2015). Referral rates in Swiss primary care with a special emphasis on reasons for encounter. Swiss Med Wkly.

[19] Swiss Federal Office of Public Health: Statistik der obligatorischen Krankenversicherung. 2018. https://www.bag.admin.ch/bag/de/home/service/zahlen-fakten/statistiken-zur-krankenversicherung/statistik-der-obligatorischen-krankenversicherung.html. Accessed 10 Feb 2018.

[20] Hostettler S, Kraft E (2017). FMH-Ärztestatistik 2016 36 175 berufstätige Ärztinnen und Ärzte. Schweiz Ärzteztg.

[21] The Commonwealth Fund: The Swiss Health Care System. 2016. https://international.commonwealthfund.org/countries/switzerland/ Accessed 01 Aug 2018.

[22] Programm für Nationale VersorgungsLeitlinien von BÄK, KBV und AWMF: Qualitätsindikatoren– Manual für Autoren. 2009. http://www.aezq.de/mdb/edocs/pdf/schriftenreihe/schriftenreihe36.pdf. Accessed 10 Feb 2018.

[23] AQUA Institut: Allgemeine Methoden. 2015. https://www.aquainstitut.de/fileadmin/aqua_de/Projekte/248_Methodenpapier/Methodenpapier_4.0.pdf. Accessed 10 Feb 2018.

[24] German College of General Practitioners and Family Physicians: S3-Leitlinien der DEGAM - der Zehnstufenplan. 2009. http://www.degam.de/id-10-stufen-plan.html. Accessed 10 Feb 2018.

[25] Huber CA, Szucs TD, Rapold R, Reich O (2013). Identifying patients with chronic conditions using pharmacy data in Switzerland: an updated mapping approach to the classification of medications. BMC Public Health.

[26] Schwabe U, Paffrath D (2016). Arzneiverordnungs-Report 2016.

[27] Holt S, Schmiedl S, Thurmann PA (2010). Potentially inappropriate medications in the elderly: the PRISCUS list. Dtsch Arztebl Int.

[28] American Geriatrics Society (2012). Beers Criteria Update Expert Panel. American Geriatrics Society updated Beers Criteria for potentially inappropriate medication use in older adults. J Am Geriatr Soc.

[29] World Health Organization: ATC/DDD Index. 2018. http://www.whocc.no/atc_ddd_index/. Accessed 10 Feb 2018.

[30] Huber CA, Brändle M, Rapold R, Reich O, Rosemann T (2016). A set of four simple performance measures reflecting adherence to guidelines predicts hospitalization: a claims-based cohort study of patients with diabetes. Patient Prefer Adherence..

[31] Huber CA, Rapold R, Brüngger B, Reich O, Rosemann T (2016). One-year adherence to oral antihyperglycemic medication and risk prediction of patient outcomes for adults with diabetes mellitus: An observational study. Medicine (Baltimore).

[32] Huber CA, Reich O, Früh M, Rosemann T (2016). Effects of Integrated Care on Disease-Related Hospitalisation and Healthcare Costs in Patients with Diabetes, Cardiovascular Diseases and Respiratory Illnesses: A Propensity-Matched Cohort Study in Switzerland. Int J Integr Care.

[33] Schweizer Forum für Intergrierte Versorgung fmc: Presentation of Schmutz DH at the Swiss national Symposium of Integrated Care2016, Bern, Switzerland. Performance-basierte Vergütung: Auch im Gesundheitswesen? 2016. https://fmc.ch/_Resources/Persistent/44cf7867c819aec35e6beac171ce7640ccf60b68/2016_Keynote_4_Daniel_H_Schmutz.pdf. Accessed 10 Feb 2018.

[34] Ebert ST, Pittet V, Cornuz J, Senn N. Development of a monitoring instrument to assess the performance of the Swiss primary care system. BMC Health Serv Res. 17(1):789. 10.1186/s12913-017-2696-z PMC5707782 29187196

[35] Herzlinger RE, Parsa-Parsi R (2004). Consumer-driven health care: lessons from Switzerland. JAMA.

[36] Helsana Group: Helsana Drug Report 2017. 2017. https://www.helsana.ch/en/helsanagroup/about-our-company/health-sciences/drug-report. Accessed 10 Feb 2018.

[37] Helsana Group: Helsana Report Ausgabenentwicklung in der Gesundheitsversorgung. 2016. https://www.helsana.ch/de/helsana-gruppe/unternehmen/gesundheitswissenschaften/ausgabenreport. Accessed 10 Feb 2018.

[38] Pohontsch NJ, Herzberg H, Joos S, Welti F, Scherer M, Blozik E (2015). The professional perspective on patient involvement in the development of quality indicators: a qualitative analysis using the example of chronic heart failure in the German health care setting. Patient Prefer Adherence..

[39] Kötter T, Schaefer FA, Scherer M, Blozik E (2013). Involving patients in quality indicator development - a systematic review. Patient Prefer Adherence.

[40] Mendelson A, Kondo K, Damberg C, Low A, Motúapuaka M, Freeman M, O'Neil M, Relevo R, Kansagara D (2017). The Effects of Pay-for-Performance Programs on Health, Health Care Use, and Processes of Care: A Systematic Review. Ann Intern Med.

[41] Ryan AM, Krinsky S, Kontopantelis E, Doran T (2016). Long-term evidence for the effect of payfor-performance in primary care on mortality in the UK: a population study. Lancet.

[42] Kötter T, Blozik E, Scherer M (2012). Methods for the guideline-based development of quality indicators--a systematic review. Implement Sci.

[43] Blozik E, Lühmann D, Scherer M, Amelung VE, Eble S, Hildebrandt H, Knieps F, Lägel R, Ozegowski S, Schlenker R-U, Sjuts R (2017). Entwicklung und Validierung von Qualitätsindikatoren für Multimorbidität (Multiqual). Innovationsfonds, Impulse für das deutsche Gesundheitswesen.

